# Immunohistochemical detection and regulation of α_5 _nicotinic acetylcholine receptor (nAChR) subunits by FoxA2 during mouse lung organogenesis

**DOI:** 10.1186/1465-9921-12-82

**Published:** 2011-06-17

**Authors:** Jason L Porter, Benjamin R Bukey, Alex J Geyer, Charles P Willnauer, Paul R Reynolds

**Affiliations:** 1 Department of Physiology and Developmental Biology, Brigham Young University, Provo, UT 84602, USA

**Keywords:** alpha 5, development, epithelium, lung, nAChR

## Abstract

**Background:**

α_5 _nicotinic acetylcholine receptor (nAChR) subunits structurally stabilize functional nAChRs in many non-neuronal tissue types. The expression of α_5 _nAChR subunits and cell-specific markers were assessed during lung morphogenesis by co-localizing immunohistochemistry from embryonic day (E) 13.5 to post natal day (PN) 20. Transcriptional control of α_5 _nAChR expression by FoxA2 and GATA-6 was determined by reporter gene assays.

**Results:**

Steady expression of α_5 _nAChR subunits was observed in distal lung epithelial cells during development while proximal lung expression significantly alternates between abundant prenatal expression, absence at PN4 and PN10, and a return to intense expression at PN20. α_5 _expression was most abundant on luminal edges of alveolar type (AT) I and ATII cells, non-ciliated Clara cells, and ciliated cells in the proximal lung at various periods of lung formation. Expression of α_5 _nAChR subunits correlated with cell differentiation and reporter gene assays suggest expression of α_5 _is regulated in part by FoxA2, with possible cooperation by GATA-6.

**Conclusions:**

Our data reveal a highly regulated temporal-spatial pattern of α_5 _nAChR subunit expression during important periods of lung morphogenesis. Due to specific regulation by FoxA2 and distinct identification of α_5 _in alveolar epithelium and Clara cells, future studies may identify possible mechanisms of cell differentiation and lung homeostasis mediated at least in part by α_5_-containing nAChRs.

## Background

Pulmonary development adheres to orchestrated processes that require precisely regulated reciprocal interactions between developing respiratory epithelium and the surrounding splanchnic mesenchyme. Proper lung development involves both spatial and temporal control of a myriad of factors including transcription factors, growth factors, cell surface receptors, and extracellular matrix constituents. Notably, lung development requires cell migration during branching morphogenesis, cell polarization, and differentiation of specialized cells along the proximal/distal pulmonary axis [1]. Diverse transcription factors and signaling proteins function in intricate signaling and regulatory mechanisms during pulmonary cell differentiation. Such important contributing molecules include FoxA2, and GATA-6 [2,3]. FoxA2 is a transcription factor prominently expressed by the lung that contains a winged helix DNA binding domain [4]. Necessary for the formation of foregut derivatives, FoxA2 functions in the differentiation of respiratory epithelium and contributes to normal branching morphogenesis and cell commitment [2]. Later in development, FoxA2 regulates several genes required for lung function after birth including surfactant proteins, TTF-1, Muc5A/C, E-cadherin and Vegfa [5-9]. GATA-6 is a zinc-finger containing transcription factor expressed by respiratory epithelial cells throughout lung morphogenesis. GATA-6 is required for specialization of bronchiolar epithelium [10] and it contributes to sacculation and alveolarization in concert with numerous other transcriptional regulators [11,12]. At precise time points, signaling involving these and other molecules mediate epithelial-mesenchymal interactions and provide signals that induce lung-specific genetic programs vital for proper pulmonary morphogenesis. Importantly, the functional contributions of critical genes during development depend on precise expression patterns that result from mechanisms initiated by signal transduction pathways. Understanding cell populations that co-express important regulatory proteins and specific cell surface receptors may identify relevant receptors that contribute to transcription factor expression and ultimate lung formation.

Neuronal nicotinic acetylcholine receptors (nAChRs) are ligand-gated cation channels that form the principal excitatory neurotransmitter receptors in the peripheral nervous system [13]. Specifically, nAChRs mediate chemical neurotransmission among neurons, ganglia, interneurons, and the motor endplate. The biology of nAChRs has expanded in recent years due to nAChR localization in several non-neuronal tissues, including the lung [14,15]. NAChRs are pentameric oligomers composed of five subunits that surround a central ion channel through which ions flow following ligand binding. Receptor subunits have been identified as either agonist binding (α_2_, α_3_, α_4_, α_6_, α_7_, α_9 _and α_10_) or structural (α_5_, β_2_, β_3 _and β_4_) [13,16]. In the current investigation, the α_5 _subunit and cell-specific markers were co-localized in the developing mouse lung by immunohistochemistry so that pulmonary cell types that express α_5 _could be identified. These studies involved well-characterized antibodies that identify non-ciliated Clara cells and ciliated epithelial cells in the proximal lung, alveolar type II (ATII) cells that secrete surfactant proteins, and alveolar type I (ATI) cells that contribute abundantly to the respiratory membrane. Because expression corresponded with differentiating lung epithelial cells influenced by FoxA2 and GATA-6, experiments were conducted in order to test the hypothesis that these important pulmonary transcription factors regulate α_5_. Although little data regarding the expression pattern and specific contributions of α_5 _nAChR subunits previously existed, identification on specific pulmonary cells is an critical first step in eventually assessing possible cholinergic signaling pathways that likely influence normal and abnormal lung formation [17].

## Methods

### Animals

C57BL/6 mice were housed and used in accordance with approved IACUC protocols at Brigham Young University. Male and female mice were mated and the discovery of a vaginal plug was identified as embryonic day (E) 0.

### Antibodies and Immunohistochemistry

A rabbit α_5 _polyclonal antibody generated against cytoplasmic epitopes was used at a dilution of 1:800 to identify α_5 _nAChR subunits in the lung during development. Immunobotting and ELISAs were used to determine the specificity of the α_5 _antibody and it was determined to be effective with tissues embedded in paraffin [18]. A rabbit polyclonal antibody against Clara Cell Secretory Protein (CCSP, Seven Hills Bioreagents, Cincinnati, OH) was used at a dilution of 1:1600. A monoclonal antibody for Fox J1 (Seven Hills BioReagents) was used at a dilution of 1:2000. ATII epithelial cells were specifically identified by staining with a rabbit anti-N-terminal proSP-C polyclonal antibody (1:1000, Seven Hills BioReagents) and ATI cells were localized via staining with a monoclonal hamster anti-mouse antibody raised against T1α at a dilution of 1:2000 (Clone 8.1.1, Developmental Studies Hybridoma Bank, Department of Biology, University of Iowa, Iowa City, IA). Immunohistochemical staining involved six mice per time point and staining for each antibody was conducted on three different slides. Immunostaining for CCSP, proSP-C, T1α, FoxJ1 and α_5 _was performed with 5-μm serial sections beginning at E18.5 because this period coincided with elevated α_5 _expression and the differentiation status of epithelial cells that express these markers [19,20]. Staining of serial sections was selected over preferred methods of dual labeling immunofluorescence because specific staining using multiple rabbit polyclonal antibodies in the same slide is not easily reproducible. Sections were deparaffinized, and rehydrated by incubation in 100%, 95%, and 70% ethanol then treated with 3% hydrogen peroxide in methanol for 15 min to quench endogenous peroxidase. Following block in 2.0% normal goat serum in PBS for 2 hr at room temperature, sections were incubated with CCSP, proSP-C, T1α, or α_5 _primary antibody at 4°C overnight. Control sections were incubated in blocking serum alone. After overnight incubation with primary antibody, all sections (including controls) were washed and positive staining was detected using biotinylated goat anti-rabbit secondary antibodies and a Vector Elite ABC kit (Vector Laboratories; Burlingame, CA). Development in nickel diaminobenzidine was followed by incubation in Tris-cobalt (which enhances antigen localization), and counterstaining was conducted with nuclear fast red. Sections were dehydrated by incubation in 70%, 95%, and 100% ethanol, washed in three changes of HistoClear (Fisher Scientific, Waltham, MA), and mounted under cover slips with mounting medium. Immunohistochemical staining for FoxJ1 was completed using a "Mouse on Mouse" monoclonal antibody kit (Vector) in accordance with the manufacturer's instructions. Individuals blinded to the antibody used initially imaged the serial sections and co-localization was determined by comparing immunolabeling of α_5 _with cells that express CCSP, FoxJ1, proSP-C, or T1α.

### Plasmids, Cells, and Reporter Gene Assays

0.85-kb of the mouse α_5 _promoter was obtained by polymerase chain reaction (PCR), ligated into a pGL4.10 reporter vector (Promega, Madison, WS) and verified by sequencing as described previously [21]. Site-directed mutagenesis of a potential FoxA2 binding site (-488) was performed by using the 0.85-kb reporter construct and the QuickChange™ Site-Directed Mutagenesis kit (Stratagene, La Jolla, CA). The sequence verified mutant reporter contained synthetic oligonucleotides for the desired mutation for FoxA2 (CATTTA→GGGGGG). Functional assays of reporter gene constructs were performed by transient transfection of Beas-2B and A549 cells using FuGENE-6 reagent (Roche, Indianapolis, IN) [21]. Beas-2B is a transformed human bronchiolar epithelial cell line and A549 is a human pulmonary adenocarcinoma cell line characteristic of ATII cells [22]. Transfections included 500 ng pRSV-βgal, 100 ng pGL4.10-0.85-kb α_5_, 100-400 ng pCMV-FoxA2 or pCMV-GATA-6 and pcDNA control vector to bring total DNA concentration to 1.4 μg. After 48 hours, plates were scraped and centrifuged, and the cleared supernatant was used for both β-gal and luciferase assays such that assays were normalized for transfection efficiency based on the β-gal activity [19]. Data presented are representative of three different experiments, all performed in triplicate.

### Statistical Analysis

Results are presented as the means ± S.D. of six replicate pools per group. Means were assessed by one and two-way analysis of variance (ANOVA). When ANOVA indicated significant differences, student t tests were used with Bonferroni correction for multiple comparisons. Results are representative and those with p values < 0.05 were considered significant.

## Results

### Temporal/spatial pattern of α_5 _expression in developing mouse lung

**Figure 1:**
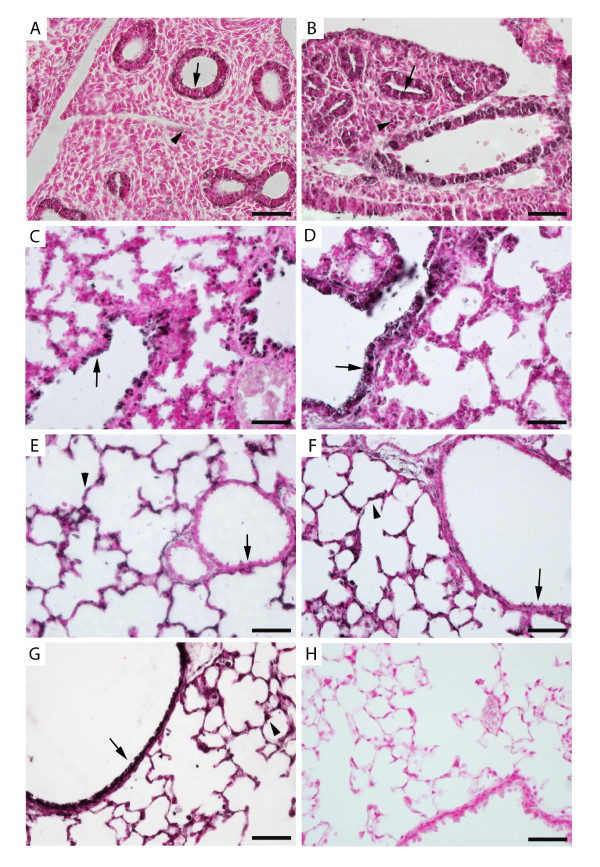
**Immunolocalization of α**_**5 **_**nAChR subunits during periods of murine lung morphogenesis**. α_5 _was primarily detected in primitive respiratory epithelium at E13.5 (A, arrow) and E15.5 (B, arrow) and only minimally detected in mesenchyme (arrowheads). During the saccular stage of lung development (E18.5, C and PN1, D), α_5 _was prominently located on respiratory epithelium in the larger airways (arrows). Expression of α_5 _in airway epithelium was diminished at PN4 (E, arrow) and PN10 (F, arrow) and common in distal lung epithelium (arrowheads). At PN20, robust expression of α_5 _was again detected throughout the proximal lung airways (G, arrow) and expression persisted in the periphery (G, arrowhead) at the completion of alveologenesis. No immunoreactivity was observed in PN20 lung sections incubated without primary antibody (H). All images are at 40X original magnification and scale bars represent 50 μm.

The distribution of α_5 _expression in mouse lung was assessed by immunohistochemistry from E13.5 to PN20. At E13.5 (Figure 1A) and E15.5 (Figure 1B), α_5 _was primarily detected in epithelial cells that comprise the primitive conducting airways of the developing lung and only sporadically expressed in mesenchyme. At E18.5 (Figure 1C), and PN1 (Figure 1D), α_5 _was predominantly expressed in proximal lung epithelial cells with diminished expression in distal lung epithelium. At PN4 (Figure 1E), α_5 _was detected in the distal lung, while staining in the conducting airways was markedly decreased. This shift in α_5 _expression from proximal to distal lung epithelium at PN1 and PN4 was also observed at PN10 (Figure 1F). At PN20 (Figure 1G), robust α_5 _expression returned to proximal lung epithelium while α_5 _localization persisted in the distal lung. No staining was observed in sections stained without primary antibody (Figure 1H).

### Association of α_5 _expression with cell-specific markers

**Figure 2:**
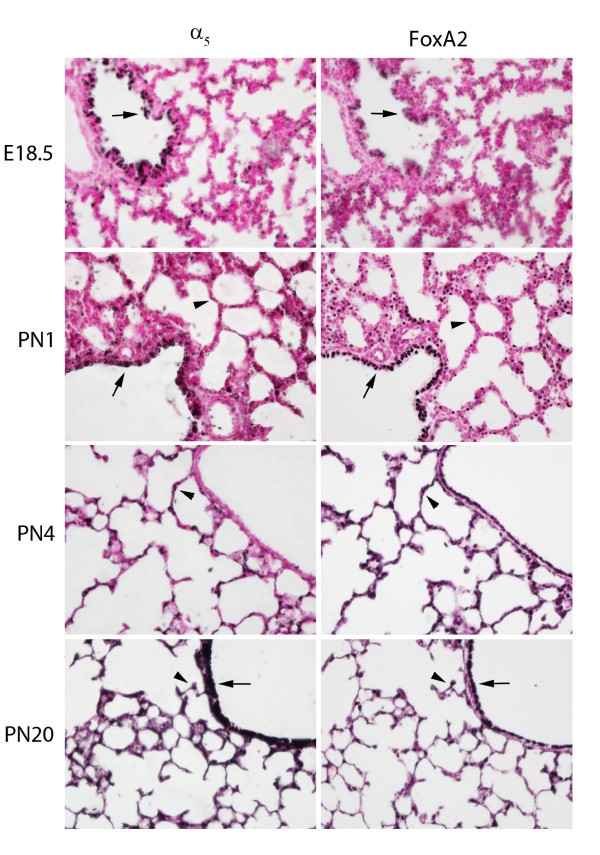
**Co-immunostaining of α**_**5 **_**nAChR subunits and FoxA2 during periods of lung development**. α_5 _(A, C, E, G) was observed in cells that also express FoxA2 (B, D, F, H). Prominent co-expression was observed in airway epithelium (arrows) at E18.5 (A, B), PN1 (C, D), and PN20 (G, H). Co-expression of α_5 _and FoxA2 was also detected in respiratory epithelium (arrowheads) at PN1 (C, D), PN4 (E, F), and PN20 (G, H).

**Figure 3:**
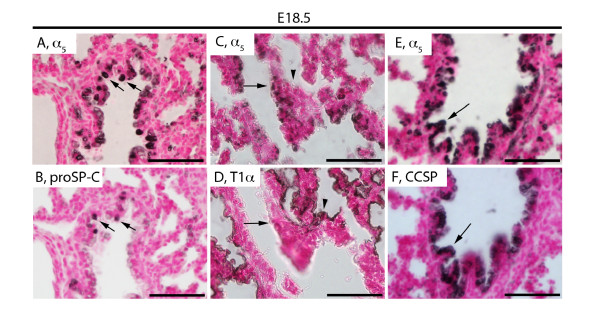
**Immunostaining of α**_**5 **_**nAChR subunits, proSP-C, T1α, and CCSP during the mid-saccular period of lung development (E18.5)**. α_5 _(A, B) was co-expressed with proSP-C (B, arrows) in most ATII cells. α_5 _was expressed in non-ATI cells in respiratory airways (C, D arrows) and poorly expressed by T1α-positive ATI cells (C, D arrowhead). α_5 _(E) was also expressed by non-ciliated Clara cells in the proximal lung as revealed by CCSP co-localization (F, arrows). Lung sections stained without primary antibodies were negative (not shown). All images are at 40X original magnification and scale bars represent 50 μm.

In order to identify specific cell populations that express α_5_, co-localizing immunohistochemistry was performed on serial sections obtained from mice at E18.5 through PN20. During the early saccular period (E18.5), α_5 _was co-expressed with FoxA2, a general marker of primitive respiratory and airway epithelium in the proximal and distal lung (Figure 2A, B). Co-expression of α_5 _and FoxA2 was also detected in proximal and distal pulmonary epithelium at PN1 (Figure 2C, D), PN4 (Figure 2E, F), and PN20 (Figure 2G, H). Expression by differentiating ATII cells at E18.5 was confirmed by co-localizing α_5 _expression with proSP-C (Figure 3A, B). Staining for T1α, an ATI-specific marker, revealed that α_5 _was not expressed by ATI cells at E18.5 (Figure 3C, D). Significant co-localization with CCSP, a marker for Clara cells in the proximal lung, was also observed at E18.5 (Figure 3E, F).

**Figure 4:**
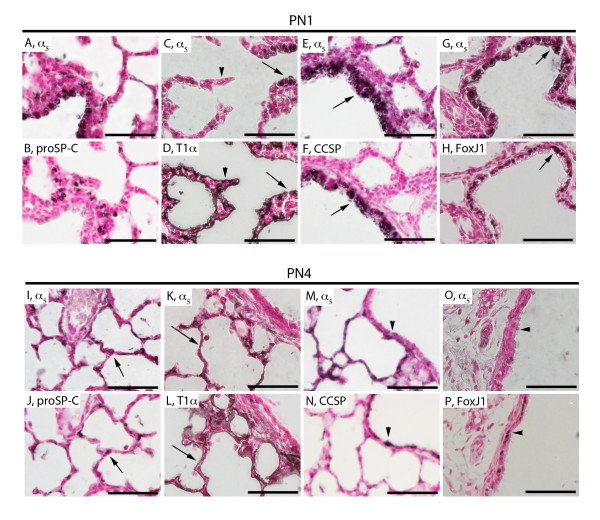
**Immunostaining of α**_**5 **_**nAChR subunits, proSP-C, T1α, CCSP, and FoxJ1 during the mid-saccular post natal period (PN1) and late saccular period (PN4) of lung development**. α_5 _(A, C) did not clearly co-localize with proSP-C expressing ATII cells (B) and was detected in some ATI cells stained with T1α (D, arrows) but not all (D, arrowhead). α_5 _expression (E, G) was abundantly detected in the proximal lung as evidenced by co-expression by CCSP-expressing Clara cells (F, arrow) and ciliated cells in the proximal airways that express FoxJ1 (H, arrow). At PN4, α_5 _(I, K) was co-expressed by ATII and ATI cells via co-localization with proSP-C (J, arrow) and T1α (L, arrow), respectively. PN4 was a period in which α_5 _expression was nearly absent in the proximal lung, therefore co-localization with CCSP in Clara cells (N, arrowhead) and FoxJ1 in ciliated cells (P, arrowhead) was poor. Lung sections stained without primary antibodies were negative (not shown). All images are at 40X original magnification and scale bars represent 50 μm.

At PN1, a period that coincides with the mid-saccular stage, α_5 _was detected in only a minority of ATII cells via proSP-C co-localization (Figure 4A, B) and ATI cells stained for T1α (Figure 4C, D). At PN1, significant detection of α_5 _in CCSP-positive Clara cells (Figure 4E, F) and cells that express FoxJ1 (Figure 4G, H), a transcription factor vital in ciliogenesis, revealed α_5 _expression in both non-ciliated and ciliated bronchiolar epithelium. At the end of the saccular period (PN4), staining for proSP-C (Figure 4I, J) and T1α (Figure 4K, L) revealed that α_5 _was expressed by ATII and ATI cells, respectively. Immunostaining with CCSP (Figure 4M, N) and FoxJ1 (Figure 4O, P) reveal that α_5 _expression is absent in non-ciliated Clara cells and ciliated epithelial cells in the proximal lung. These data suggest that α_5 _expression is chiefly identified on Clara cells in the proximal lung at PN1 and on ATII and ATI cells in the distal lung at PN4.

**Figure 5:**
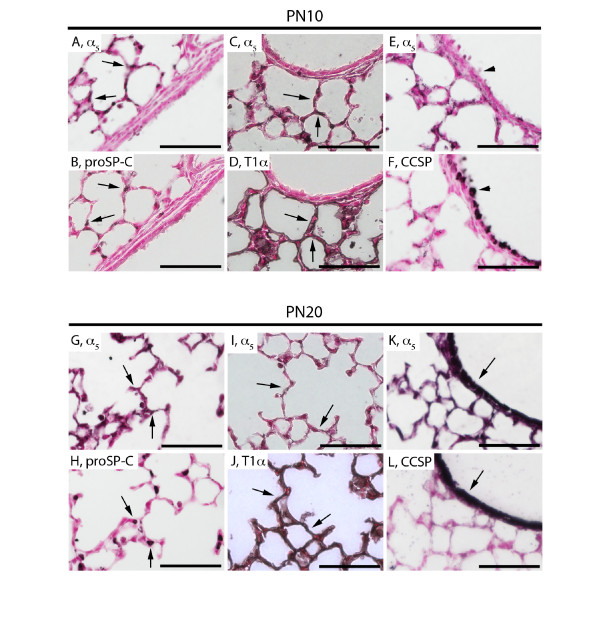
**Immunostaining of α**_**5 **_**nAChR subunits, proSP-C, T1α, and CCSP during the mid-alveolar (PN10) and near the conclusion of the alveolar period (PN20) of lung development**. α_5 _(A, C) expression at PN10 persisted in distal lung ATII cells that express proSP-C (B, arrows) and ATI cells that express T1α (D, arrows). This period also coincided with undetectable α_5 _expression in the proximal lung (E) revealing no co-localization with CCSP (F, arrowhead). At PN20, α_5 _(G, I) expression remained detectable in ATII cells that express proSP-C (H, arrows) and ATI cells that express T1α (J, arrows). This period agreed with a return to robust α_5 _expression in the proximal lung (K, arrow), most notably by Clara cells that express CCSP (L, arrow). Lung sections stained without primary antibodies were negative (not shown). All images are at 40X original magnification and scale bars represent 50 μm.

During the mid-alveolar stage of lung development (PN10), staining performed with proSP-C revealed that most but not all ATII cells express α_5 _(Figure 5A, B) and staining for T1α demonstrated that ATI cells express α_5 _(Figure 5C, D). As was observed at PN4, CCSP co-immunostaining revealed no detectable α_5 _expression in proximal lung epithelium (Figure 5E, F). A significant general observation near the end of the alveolar period (PN20) was that α_5 _staining markedly returns to the large airways at the conclusion of alveologenesis. Co-localization with proSP-C-positive ATII cells (Figure 5G, H) and T1α-positive ATI cells (Figure 5I, J) confirmed α_5 _expression by alveolar epithelial cells. Staining for CCSP also revealed markedly increased α_5 _expression by proximal bronchiolar epithelium (Figure 5K, L).

### Transcriptional Control of α_5 _in pulmonary epithelium by FoxA2 and GATA-6

**Figure 6:**
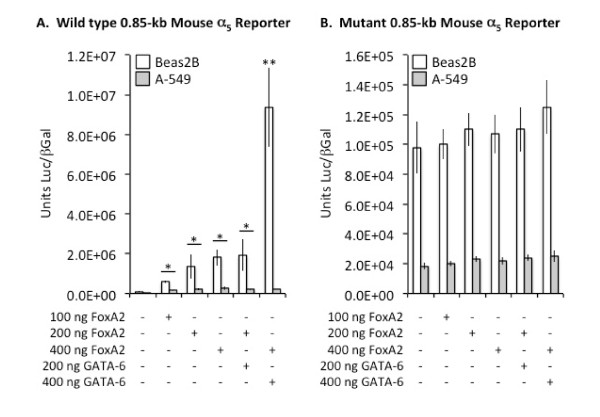
**FoxA2 induced α**_**5 **_**transcription in bronchiolar and alveolar epithelial cell lines**. FoxA2 dose-dependently induced α_5 _transcription by acting on a 0.85-kb α_5 _reporter in Beas-2B and A549 cells (A). FoxA2 and GATA-6 also cooperated to induce a highly significant increase in α_5 _transcription in Beas-2B cells (A) but did not elicit a similar increase in A549 cells. Mutagenesis of a single putative FoxA2 response element completely eliminated FoxA2-mediated increases in α_5 _transcription and inhibited FoxA2-GATA-6 cooperation in the regulation of α_5 _gene expression (B). Significant differences in luciferase levels compared to reporter alone are noted at P ≤ 0.05 (*) and P ≤ 0.01 (**).

Because the expression pattern of α_5 _nAChR subunits coincided with differentiating pulmonary epithelial cells in both the proximal and distal lung compartments, we sought to determine the regulatory effects of FoxA2 and GATA-6 on α_5 _transcription. Reporter gene assays in bronchiolar Beas-2B cells revealed that α_5 _transcription is significantly increased by FoxA2 (Figure 6A). While increasing concentrations of GATA-6 alone had no effect on α_5 _transcription (not shown), when combined, both FoxA2 and GATA-6 synergistically induced elevated α_5 _transcription in Beas-2B cells (Figure 6A). In alveolar type II-like A549 cells, FoxA2 also significantly increased α_5 _transcription in a dose dependent manner (Figure 6A); however, GATA-6 had no measurable effect, either individually (not shown) or in combination with FoxA2 (Figure 6A). Mutagenesis of a single putative FoxA2 response element resulted in complete ablation of FoxA2 transcriptional activation of α_5 _expression in both Beas-2B and A549 cells (Figure 6B). Furthermore, possible interactions between FoxA2 and GATA-6 in the regulation of the α_5 _gene were also inhibited when the possible FoxA2 response element was removed (Figure 6B).

## Discussion and Conclusions

Immunostaining for α_5 _nAChR subunits revealed an interesting pattern of expression during periods of lung formation. Utilization of antibodies for cell-specific markers demonstrated that various pulmonary epithelial cell populations express α_5 _subunits during distinct periods of lung organogenesis. An intriguing discovery was that α_5 _expression experienced profound shifts between proximal and distal lung epithelial cells during perinatal milestones. For example, conducting airway epithelial cell expression persisted throughout embryonic and post-natal lung morphogenesis except at PN4 and PN10, a period that is characterized by parenchymal differentiation in the alveolar period of lung formation. Furthermore, staining in the distal lung was evident at E18.5, but noticeably diminished at PN1. Precise regulation of α_5 _nAChR subunits that stabilize a subset of functional pentameric nAChRs suggests the possibility that nAChR-mediated signaling may participate in specific epithelial cell differentiation trajectories.

Because immunolocalization of α_5 _was primarily detected on luminal membranes of various epithelial cell populations, it is likely that α_5 _subunits accumulate on the apical surface in order to contribute to functional nAChRs. Furthermore, intense expression at PN20, a period that coincides with the final stages of alveologenesis occurring from PN5-30 in the mouse [23], suggests α_5 _may function in the maintenance of the post-natal lung. It is possible that α_5_-containing nAChRs function *in utero *by binding ligand and inducing signal transduction required during embryonic development. These possibilities are supported by previous research that identify functional nAChRs in various lung epithelial cells [24-26]. Because α_5 _co-localizes with multiple transcription factors essential in lung development such as TTF-1 [21], FoxA2, and GATA-6, our data clearly suggest that α_5_-containing nAChRs may function in mediating paracrine communication between respiratory epithelial cell populations.

Previous work in our laboratory revealed that α_5 _is co-expressed with TTF-1 [21]. TTF-1 is a molecule expressed in lung periphery during early pulmonary development and critical in regulating the expression of genes necessary for branching morphogenesis and cell differentiation [5,27,28]. The importance of TTF-1 is demonstrated by severe hypoplastic lung malformation observed in mice lacking TTF-1 [29]. The concept that α_5 _and TTF-1 cooperate in signaling is supported by site-directed mutagenesis data from our lab that reveal TTF-1 transcriptionally regulates α_5 _expression via binding to specific TTF-1 response elements located in the proximal α_5 _promoter [21]. Co-localization of α_5 _with cells that express FoxA2 also increases the likelihood that α_5 _may function in pulmonary cell differentiation. FoxA2 is a protein that contains a winged double helix DNA binding domain [4] and it is expressed in an overlapping pattern with TTF-1 [30]. FoxA2 directly and in combination with GATA-6 influences respiratory epithelial cell differentiation [2] and it significantly regulates the promoters of α_5 _(Figure 6) and TTF-1 [6]*in vitro*. Therefore, it is possible that TTF-1 and FoxA2 co-activate multiple genes that potentially contribute to cell differentiation pathways, including α_5 _nAChR subunits. Specifically relevant to the current study is the discovery that a single putative FoxA2 binding site exists in the proximal α_5 _promoter and that plausible GATA-6 binding sites are absent. This suggests that possible transactivation by GATA-6 is likely mediated by other DNA-binding proteins such as FoxA2. Importantly, our research may clarify additional functions of TTF-1 and FoxA2 that already are known to interact in the regulation of genes critical to lung function, including CCSP, surfactant proteins, growth factors, and Vegfa/Vegfr2 interactions essential in vasculogenesis [30].

Despite clear localization of α_5 _with TTF-1 [21] and FoxA2 (Figure 2), as well as cell-specific markers such as CCSP and proSP-C, co-localization was not completely identical. For instance, epithelium specific transcription factors such as TTF-1 and FoxA2 have not been functionally characterized as factors that control mesenchymal gene expression. Therefore, α_5 _expression is likely controlled by the activity of many overlapping factors such as TTF-1, FoxA2, Gata-6, NF-1, RAR, and AP-1, and the precise pattern of α_5 _expression is plausibly influenced by complex interplay between competing and redundant activators [31].

At PN1, α_5 _co-localized with FoxJ1, a nuclear protein vital in the regulation of multiple genes necessary for ciliogenesis in ciliated cells resident in conducting airways [32,33]. The fact that co-localization with FoxJ1 was not observed after PN1 reveals that differentiated ciliated bronchiolar epithelial cells may not require α_5 _subunit expression at the onset of alveologenesis. Once α_5 _expression returned to the proximal lung at PN20, co-localization was most prominent in non-ciliated Clara cells, suggesting possible roles for α_5_-containing nAChR signaling in protective functions and regenerative capacity mediated by Clara cells in the conducting airways [34].

Cell differentiation and proper organ formation involves complex interrelated mechanisms that can be deleteriously altered when noxious ligands are present. For instance, the availability of nicotine during important periods of lung development can affect normal lung developmental programs. Our data reveal that α_5_-containing nAChRs are expressed on ATI, ATII, Clara and ciliated epithelial cells, all of which are affected when nicotine crosses the placenta during development. Specifically, exposure to cigarette smoke during pregnancy adversely affects lung development by significantly reducing branching morphogenesis [35], increasing rates of respiratory illness [36], irreversibly altering pulmonary function [37], and permanently obstructing proximal lung airways [38]. Important research performed by Carlisle et al. involving the characterization of nAChR subunits in the lungs of never smokers, ex-smokers, and active smokers revealed altered nAChR expression depending on smoke status [39]. At the protein level, α_5 _is up-regulated by pulmonary epithelium in response to chronic nicotine exposure and there were fewer never smokers that express α_5 _protein compared to active smokers (p < 0.05) [39]. Our studies demonstrate that α_5_-containing nAChRs are expressed in populations of epithelial cells during normal lung development; however, α_5_-containing nAChRs may also function during morphological perturbation of the lung when noxious ligands such as nicotine are present.

In summary, cellular expression of α_5 _nAChR subunits varies during lung morphogenesis. α_5 _is expressed in distal lung epithelial cells during development while proximal lung expression markedly alternates between intense prenatal expression, absence at PN4 and PN10, and a return to pronounced expression at PN20. α_5 _expression was observed in differentiating ATI and ATII cells and proximal Clara and ciliated cells at specific time points of organ formation, and adult expression is consistently identified in respiratory epithelium and Clara cells. The data suggest that expression of α_5_-containing nAChRs is specifically controlled during lung morphogenesis and that regulation occurs in part by FoxA2 and Gata-6. However, the precise functions of α_5 _in the maturing lung are still unclear. Experiments aimed at discovering possible roles for α_5_, including gene targeting in cells that persistently express or block α_5 _both during and after morphogenesis, are underway and should provide additional clues into the biology of α_5 _subunits.

## Competing interests

The authors declare that they have no competing interests.

## Authors' contributions

JLP, BRB, and AJG performed immunohistochemistry and assisted in manuscript preparation. CPW generated plasmids and performed the *in vitro *reporter gene assays. PRR conceived of the study and supervised in its implementation, interpretation, and writing. All authors approved of the final manuscript.
